# Enhancing Sensory Quality of Coffee: The Impact of Fermentation Techniques on *Coffea arabica* cv. Catiguá MG2

**DOI:** 10.3390/foods13050653

**Published:** 2024-02-21

**Authors:** Lívia C. F. Silva, Paulo V. R. Pereira, Marcelo A. D. da Cruz, Gisele X. R. Costa, Renata A. R. Rocha, Pedro L. L. Bertarini, Laurence R. do Amaral, Matheus S. Gomes, Líbia D. Santos

**Affiliations:** 1 Biotechnology Institute, University Federal of Uberlândia, Patos de Minas 38702-178, MG, Brazil; livia.fidelis@ufu.br (L.C.F.S.); paulo.rocha@ufu.br (P.V.R.P.); marcelo.antonio@ufu.br (M.A.D.d.C.); renata.reis@ufu.br (R.A.R.R.); matheusgomes@ufu.br (M.S.G.); 2 Faculty of Chemical Engineering, Federal University of Uberlândia, Patos de Minas 38702-178, MG, Brazil; gisele.ribeiro@ufu.br; 3 Faculty of Electrical Engineering, Federal University of Uberlândia, Patos de Minas 38702-178, MG, Brazil; bertarini@ufu.br; 4 Faculty of Computation, Federal University of Uberlândia, Patos de Minas 38702-178, MG, Brazil; laurence@ufu.br

**Keywords:** coffee fermentation, artificial intelligence, specialty coffees

## Abstract

Fermentation, a critical post-harvest process, can be strategically manipulated to augment coffee quality. This enhancement is achieved through the activity of microorganisms, which generate metabolites instrumental in the formation of distinct sensory profiles. This study investigated the impact of different fermentation methods on the quality of coffee beverages, specifically utilizing the Catiguá MG2 variety. The experimental setup involved fermenting the coffee in 200 L bioreactors, employing both natural and pulped coffee beans. The fermentation process utilized was self-induced anaerobic fermentation (SIAF), conducted in either a solid-state or submerged medium over a 96 h period. Analytical sampling was conducted initially and at 24 h intervals thereafter to quantify the concentration of sugars, alcohols, and organic acids. Sensory evaluation was performed using the established protocols of the Specialty Coffee Association (SCA). The outcomes of this investigation reveal that fermentation substantially enhances the quality of coffee, with each treatment protocol yielding divergent profiles of acids and alcohols, thereby influencing the sensory characteristics of the resulting beverage. Notably, superior quality beverages were produced from naturally processed coffee subjected to solid-state fermentation for durations exceeding 24 h. These findings underscore the significant influence of fermentation techniques and duration on the sensory attributes and overall quality of coffee.

## 1. Introduction

Coffee is one of the most consumed beverages globally, ranking as the second-most traded commodity in the world, only surpassed by oil [1,2]. For the coffee year 2022/23, global coffee production (Arabica and Robusta) is estimated to have reached approximately 10.368 billion tons, an increase of 396.0 million tons compared to the previous year [3]. Brazil stands out as the leading producer and exporter of coffee worldwide, with its production chain involving around 2000 municipalities and directly impacting over 8 million Brazilians. Among the cultivated varieties, the most prevalent are *Coffea arabica* (Arabica coffee) and *Coffea canephora* (Robusta coffee), with the former being recognized for producing beverages of superior quality, characterized by smoother, richer sensory attributes in flavor and aroma [4].

The demand of coffee consumers has become increasingly refined, focusing more on the physical and sensory quality of the beans. This phenomenon is directly reflected in the specialty coffee market, which is distinguished by its excellence in beverage quality [5]. Changes in consumer preferences are reshaping the coffee market, boosting the specialty coffee segment, and influencing the formation of coffee prices in the global market, where the added value of the product is a significant competitive differentiator [6,7].

As defined by the Specialty Coffee Association (SCA), the term “specialty coffee” refers to a beverage of superior quality, characterized by beans free from impurities and defects, and endowed with distinct sensory characteristics, scored above 80 on a rating scale. These coffees are primarily distinguished by sensory attributes such as fragrance/aroma, uniformity, absence of defects, sweetness, flavor, acidity, body, aftertaste, balance, and final note, assessed according to standardized methodologies by the SCA. The classification of specialty coffees involves sensory analysis conducted by certified Q-graders using samples of roasted coffee following the SCA protocol, the globally recognized methodology for defining specialty coffee in the international coffee market [8,9,10]. In addition, besides intrinsic quality, specialty coffees must have certified traceability and meet sustainability criteria, encompassing the optimization of environmental, social, and economic conditions at all production stages [11].

The quality of coffee and its sensory perception are influenced both directly and indirectly by a myriad of factors, including environmental (temperature, humidity, altitude, latitude, solar radiation, water availability, and soil characteristics), genetic (species, plant variety, soil, and fruit microbiota), and technological (management practices, post-harvest technologies, fermentation methods, drying, and storage). Each of these factors plays a crucial role, uniquely influencing the quality of the final beverage [12]. The growth of the specialty coffee market has encouraged producers to seek technological solutions linked to genetic and environmental factors to enhance coffee quality, aiming to add flavor and value to the product. These improvements can be implemented at various stages of the production chain, from cultivation to storage. Post-harvest management processes have significantly evolved over the years, and the use of self-induced anaerobic fermentation (SIAF) has emerged as a technological innovation in the production of specialty coffees with distinctive sensory attributes [13,14].

Fermentation is a biological process in which diverse groups of microorganisms degrade carbohydrates and other organic compounds present in the mucilaginous mesocarp of the fruit. Coffee beans absorb these compounds produced during fermentation, which transform into flavor precursors, contributing to the development of novel sensory profiles [15,16]. However, being a complex process involving a wide variety of microorganisms and enzymes, it requires a thorough understanding and mastery by producers [10,12,17]. During fermentation, parameters such as pH, temperature, and fermentation time can be controlled to modulate the process, resulting in coffees with diverse flavors and aromas. This makes fermentation a promising target for the production of specialty coffees [17,18,19]. Fermentation can occur with coffee processed naturally (with skin), pulped (de-skinned using water under pressure), partially demucilaged (partial mucilage removal using water under pressure), and washed (100% demucilaged), either using water (submerged) or without (solid state). Each method yields different sensory profiles in the beverage [20,21].

The *Coffea arabica* L. cv. Catiguá MG2 (Timor Hybrid UFV 440-10 x Catuaí Amarelo IAC 86) is a cultivar from the Genetic Improvement Program of the Empresa de Pesquisa Agropecuária de Minas Gerais (EPAMIG), developed to be resistant to Rust (*Hemileia vastatrix*) and showing great potential to produce specialty coffees [22]. Rust is the main disease affecting coffee trees; the annual loss of coffee production in Brazil as a result of rust is very high, and the use of cultivars resistant to this fungus is the most efficient control measure to overcome this problem.

This cultivar presents unique characteristics as a small tree: plagiotropic branches having short internodes, abundant secondary branching, and new leaves of light bronze color, and its large fruit turn red when ripe. Its average productivity, in bags of processed coffee per hectare, is 42.3 in Minas Gerais. As well as having the desirable characteristic of being resistant to rust, it has been recommended for the production of specialty coffees due to its high cup quality [23]. This variety was champion of the Coffee Of The Year competition—year 2021, in the natural category representing the Santiago farm, where this study was carried out.

Considering the importance of the coffee production chain in both the global and Brazilian contexts, the growing demand for specialty coffees, the desirable characteristics of the Catiguá MG2 variety, and the lack of information on factors influencing the final quality of coffee, this study aimed to evaluate in detail different protocols of self-induced fermentation of the *Coffea arabica* L. cv. Catiguá MG2 for producing specialty coffees. Variables such as coffee processing (natural and pulped coffee), water addition, pH, and fermentation time were considered to be correlated with the final physicochemical and sensory quality of the beverage in the cup based on the SCA protocol.

## 2. Materials and Methods

### 2.1. Experimental Design and Fermentation

The study utilized the *Coffea arabica* L. cv. Catiguá MG2 cultivar, planted at Santiago Farm, located at Presidente Olegário in the Alto Paranaíba region—latitude 18°32′37″, altitude 1048 m, and longitude 46°18′29″—in the state of Minas Gerais, Brazil. Coffee samples were mechanically harvested in the 2021/2022 season, utilizing both natural and pulped coffee fruit. Following harvest, the collected coffee fruit were washed, and half of them were pulped according to the farm’s protocols to obtain pulped coffee [24]. The samples were divided into experimental groups based on their fermentation time, varying at 0, 24, 48, 72, and 96 h, and subjected to self-induced fermentation in a solid state (without water addition) and submerged state (with 30% *v*/*v* water addition). The bioreactors were placed in a sunny environment for the entire fermentation time. Natural and pulped coffee fruit that did not undergo SIAF and went directly to the drying process on a raised drying bed are termed as control. The bioreactor model used in the experiment was made of non-toxic polypropylene, with a 200 L capacity, previously sanitized with water and 0.3% peracetic acid [25]. Each treatment was represented by a single sacrificial bioreactor with a capacity of 200 L. We used sacrificial bioreactors so as not to disturb the system with pH measurements and sample collection. In this way, four bioreactors were used for each fermentation condition (natural, peeled, solid-state, and submerged) and for each time evaluated (24, 48, 72, and 96 h), totaling 16 bioreactors. Samples were collected in triplicate at different points in the bioreactor after homogenizing the contents.

Each bioreactor was coupled with a data acquisition system developed by our research group at UFU, Campus Patos de Minas for collecting and storing temperature data of the fermentative medium intermittently throughout the fermentation process. A sensor was placed inside the bioreactor, and its data were sent to a microcontroller to process and store these data on a mini-SD card. All bioreactors were hermetically sealed and equipped with an S-type Airlock with a seal for gas exchange. After fermentation, the fruit were immediately washed to halt the process and subjected to drying at room temperature on a raised drying bed until they reached 11% moisture content. Once dried, the coffees were stored for a resting period of at least 30 days and then processed and classified.

### 2.2. Physical–Chemical Characterization

At the onset of the experiments, the percentage of unripe (green) fruit in the coffee batch was determined using image processing techniques via Scilab software version 2023.1.0. For this purpose, 10 images containing various coffee fruit were captured as they were placed in the bioreactor. Utilizing pattern recognition algorithms, each fruit was identified in the images, followed by a color thresholding method to classify each fruit as green or ripe. A fruit was classified as green if the majority of its pixels were of a green or greenish hue. Consequently, the percentage of green fruit in the image was calculated as the ratio of fruit identified as green to the total number of fruit identified in the image.

At the initial time and every 24 h during the fermentation process, pH and temperature were measured using an Akso^®^ SX716 pH meter. Fruit samples were collected in triplicate for subsequent analyses of moisture content and quantification of acids, alcohols, and sugars. Sacrificial bioreactors were employed to ensure that sample collection and measurements did not disrupt the ongoing fermentation systems. Both initial and fermented coffee fruit were characterized for moisture and ash content, in accordance with methodologies outlined by the Association of Official Analytical Chemists [26].

Organic acids (malic, lactic, acetic, butyric, propionic, citric, and succinic), alcohols (glycerol, ethanol), and sugars (sucrose, fructose, and glucose) were analyzed using a high-performance liquid chromatography (HPLC) system. Fruit samples from the initial time and those collected during fermentations were prepared using MilliQ water as per [27] with modifications: 10 g of fruit was mixed with 100 mL of deionized water and blended in a domestic blender (Oster 1400 W) for 2 min. The mixture was filtered through two layers of organza polypropylene and then centrifuged in a Heal Force Neofuge 18R at 13,000 rpm for 15 min at 17 °C. The supernatant was collected and filtered through a 0.22-micron nylon filter prior to injection into the Shimadzu liquid chromatography system (Shimadzu Corp., Kyoto, Japan). The extracted and filtered samples were injected into a Shimadzu HPLC system, model LC-20A Prominence, using a SUPELCOGEL C-610H column (30 mm × 7.8 mm). The mobile phase consisted of a 0.1% aqueous phosphoric acid solution, with an eluent flow rate of 0.5 mL/min, oven temperature of 32 °C, run duration of 35 min, and injection volume of 20.0 μL. Sugar and ethanol concentrations were determined using a refractive index detector, while acids were detected using a photodiode array (PDA) detector at 210 nm. Results were processed using LC-Solutions software based on their respective calibration curves.

For the green beans, texture profile analysis (TPA) was conducted using a TA-XT2i Stable Micro System texturometer, determining parameters of force and hardness. A cylindrical aluminum probe with a diameter of 25 mm, macro TPA—AIBCAKE 2, was employed. Data were collected via the “Texture Expert for Windows” software, version 1.20 (Stable Micro Systems). During testing, the probe was positioned 40 mm from the sample, with a return speed of 30 mm/second and a contact force of 30 g.

### 2.3. Sensory Analysis

Coffee samples from both control and fermented batches were prepared according to the methodology prescribed by the Specialty Coffee Association (SCA). The coffee beans were roasted to a medium level, according to the Agtron Scale, based on a standard roasting curve to ensure comparability among samples. Around 3 kg of each green coffee sample was roasted in an Atilla GOLDEN PLUS 10 roaster. The roasting temperature was kept between 187–195 °C for approximately 10 min. After roasting, a panel comprising five trained coffee experts, each holding a Q-Grader Coffee Certificate, was tasked with evaluating the samples. The sensory assessment was conducted in accordance with SCA standards, which establish the use of 8.25 g of coffee for infusion in 150 mL of mineral water (pH = 6.52) at a temperature of 93 °C, ground immediately before cupping, with a standard size of 20 mesh and encompass the evaluation of ten sensory attributes: aroma, sweetness, flavor, body, uniformity, balance, cleanliness, acidity, and aftertaste. Coffees scoring above 80 points on this scale were categorized as specialty coffees [8].

### 2.4. Data Analysis

The experiment was conducted using a completely randomized design (CRD), comprising 18 treatments, and all measurements and samples were taken with three replications each. Consequently, the effects of the treatments and fermentation duration were analyzed independently. The Shapiro–Wilk test was utilized to assess the normality of data residuals and the homogeneity of variances. Non-normal data were transformed using the formula (x + 0.5)^0.5^ to satisfy the principle of normality. Comparative analysis of mean values across all tests was performed using ANOVA followed by Tukey’s test in MiniTab^®^ version 17.1.0 (Minitab, Inc., State College, PA, USA), considering *p*-values less than 0.05 as statistically significant. The averages of the values obtained for pH, texture, sugar consumption and production of acids and alcohols were used to assess whether there was a significant difference between the treatments. Comparisons were made to assess whether there was a difference for the same treatment over the fermentation time (24, 48, 72, and 96 h compared to time zero) and whether there was a difference between the different treatments with the same fermentation process time.

For multivariate analyses, the software Past version 4.03 [28] was employed. Decision trees were generated to identify the root attributes with the highest accuracy for classifying subgroups. MATLAB software version 2023 A facilitated the evaluation of these decision trees based on: (i) characteristics of the experimental design; (ii) sensory evaluation results. The accuracy of the decision trees was ascertained using the “full-training-cross-validation” (FTCV) method, wherein all instances in the dataset are concurrently used for training and testing. The FTCV output is expressed as a percentage of the errors encountered.

## 3. Results and Discussion

### 3.1. Dynamics of Temperature and pH during Fermentation

**Figure 1:**
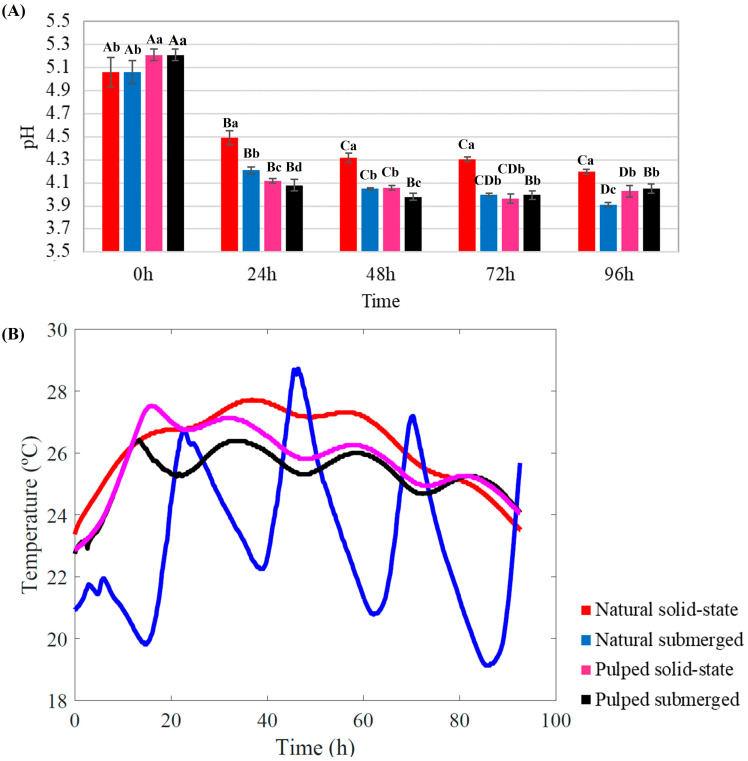
Monitoring of pH (**A**) and temperature (**B**) during the 96 h fermentation process of Catiguá MG2 coffee. The treatments evaluated were natural and pulped coffee fruit subjected to SIAF in solid-state and submerged processes. Significant statistical differences (*p* < 0.05) within the same treatment at different fermentation times are indicated by different uppercase letters above the bars, while lowercase letters indicate significant differences between different treatments in the same fermentation period.

The longitudinal changes in pH and temperature for all treatments throughout the fermentation process are depicted in Figure 1.

Throughout the fermentation process, a consistent decrease in pH was observed for all treatments. Initial pH readings for natural coffee were lower than those for pulped coffee (5.06 and 5.21, respectively) (*p* < 0.05). After 24 h of fermentation, all the treatments exhibited significant pH reduction (*p* < 0.05). At the conclusion of the 96 h SIAF, the highest pH was recorded for natural coffee in solid-state (4.20), while the lowest was observed for natural coffee submerged (3.91) among the four treatments (*p* < 0.05) (Figure 1A).

During fermentation, bacteria and yeast metabolize the sugars present in the coffee mucilage, leading to acid production and a consequent decrease in pH from 5.5–6.0 to 3.5–4.0 [10]. This pH reduction can result in more citrus and acidic flavors, which are generally desirable in coffee. However, careful monitoring of this parameter is crucial; excessively low pH levels can lead to overly acidic or even unpleasant flavors, making pH a critical control point in the production of specialty coffees [27,29].

The temperature within the bioreactor is influenced by ambient conditions and increases over the course of fermentation due to microbial activity [30]. The temperature profile within the bioreactors over the 96 h fermentation period varied between treatments. Both pulped treatments exhibited a similar profile, with an initial temperature of 23 °C. After the first 20 h, the temperature increased to approximately 28 °C for the solid-state and 26 °C for the submerged treatment. Between 20 and 80 h, a slight fluctuation around 26 °C was observed, followed by a decrease after 80 h. The natural coffee in solid-state treatment followed a similar pattern but reached and maintained higher temperatures compared to the pulped coffee treatments (28 °C) (Figure 1B).

The most distinct temperature profile among the treatments was observed in the natural coffee submerged process. It not only started at a lower initial temperature of 22 °C but also exhibited considerable fluctuations over time, reaching a minimum of approximately 17 °C and a maximum of over 28 °C (Figure 1B).

The metabolic activity of microorganisms during fermentation is a chemical reaction that releases energy. As these organisms consume sugars for energy, heat is released as a byproduct, contributing to the rise in temperature within the fermentation environment. As fermentation progresses, the microbial population may increase, leading to heightened metabolic activity and potentially intensifying the heat generated during fermentation. The literature suggests that the stabilization of or decrease in temperature during coffee fermentation can serve as an indicator to determine the end of the fermentation process [30,31,32].

The experiment was conducted in an open environment, with all bioreactors exposed to ambient temperature and solar irradiance. All treatments experienced an increase in temperature during fermentation, subject to fluctuations attributable to changes in external environmental temperatures. However, the temperature profile of the natural coffee submerged process was markedly different. This deviation could be associated with the presence of water in the treatment. Although the pulped submerged treatment also contained the same amount of water, the remaining mucilage in the coffee mixed with the water, increasing the medium’s viscosity and altering its properties, unlike the treatment with whole coffee beans (natural). Water’s thermal insulating properties, such as its high heat capacity, play a significant role here. Heat capacity is the amount of thermal energy required to change the temperature of a substance by a certain amount, causing water to heat and cool slower than other substances. This phenomenon explains the significant temperature oscillations observed in the natural coffee submerged process, in which the water warmed during the day and cooled at night, preventing a constant temperature [33] (Figure 1B).

Temperature plays a crucial role in fermentation, directly affecting microbial development and consequently influencing the flavor profile, aroma, and other characteristics of the final product. For coffee fermentation, the ideal temperature range is typically between 25 °C and 30 °C, where the microorganisms responsible for fermentation, such as bacteria and yeasts, are most active. This activity influences the development of specific flavors. Temperature, along with pH and fermentation time, are key parameters in monitoring the fermentation process. Prolonged fermentations at high temperatures and low pH can lead to the growth of undesirable microorganisms and the production of metabolites detrimental to coffee quality. Similarly, slow fermentations at lower temperatures may not result in sensory profiles that enhance the product’s flavor [20,34].

### 3.2. Green Fruit Percentage and Texture Analysis

The image processing analysis revealed that the initial sample of natural Catiguá coffee, harvested directly from the farm, contained approximately 6% green fruit. This level is considered acceptable for the fermentation of coffee aimed at producing specialty coffees. Green fruit have lower sugar and soluble compound contents than ripe fruit, which can limit the metabolic activity of microorganisms involved in fermentation. Furthermore, fermenting green fruit may result in the development of unpleasant and undesirable flavors [35,36].

Following fermentation, drying, milling, and grading of the coffees, a texture analysis of the beans was conducted, focusing on the parameters of rupture strength and hardness. The texture of fermented coffee beans can be influenced by various factors, including the processing method, fermentation duration, grain quality, and other post-harvest management aspects, which directly impact grain quality. A bean with lower rupture strength and hardness, i.e., reduced brittleness, can become more fragile, leading to losses during the drying, milling, transport, and roasting stages. No significant difference (*p* > 0.05) was observed between the treatments for either parameter, indicating that the fermentation processes do not alter the texture of the coffee bean immediately post-milling (Table S1).

### 3.3. Sugar Consumption and Production of Acids and Alcohols

**Figure 2:**
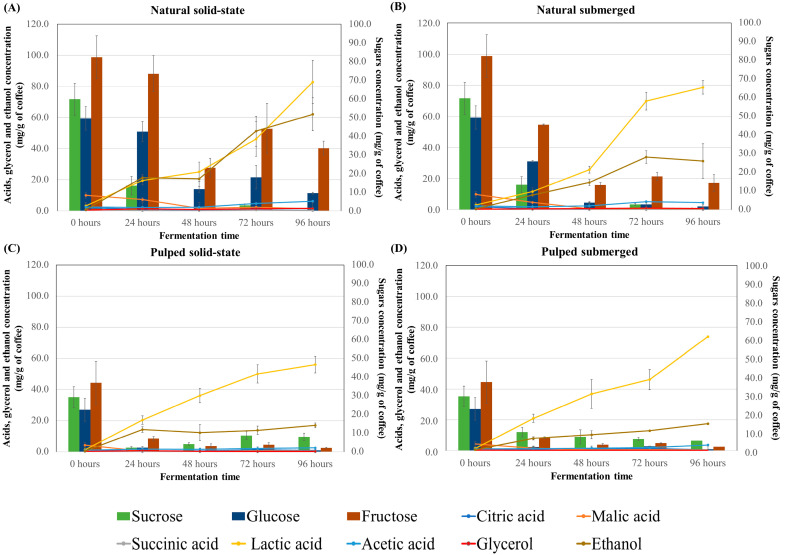
Sugar consumption profile and production of acids and alcohols during the 96 h fermentation process of Catiguá MG2 coffee (data presented in mg/g of dry coffee mass). The treatments evaluated were natural coffee in solid-state process (**A**), natural coffee in submerged process (**B**), pulped coffee in solid-state process (**C**), and pulped coffee in submerged process (**D**).

In coffee fermentation, the consumption of sugars and the production of acids are interconnected processes crucial to the development of the final coffee flavor profile. The results for sugar consumption (sucrose, glucose, and fructose) and production of acids (citric, malic, succinic, lactic, and acetic) and alcohols (glycerol and ethanol) during the fermentation process of different treatments are presented in Figure 2. At the initial timepoint, fructose was the most abundant sugar in both natural and pulped coffees (82.3 and 37.03 mg/g of coffee, respectively), followed by sucrose (59.75 mg/g for natural and 29.40 mg/g for pulped) and glucose (49.47 mg/g for natural and 22.57 mg/g for pulped). The natural coffee samples had a higher sugar content compared to the pulped ones, as part of the mucilage, which contains sugars, is removed in the pulping process.

In natural coffee treatments, sucrose was initially hydrolyzed, and by 24 h, only traces of this sugar remained (14.12 mg/g of coffee). Fructose, while not completely consumed, showed a reduction of approximately 70% from its initial amount. Glucose was also metabolized by the microorganisms, albeit at a slower rate, and some residual glucose was observed in the natural coffee in solid-state process after 96 h (9.36 mg/g of coffee).

The consumption profiles for pulped coffee treatments differed, with glucose being consumed first, followed by a gradual utilization of sucrose and fructose. After 96 h, these sugars were still detectable, but in much smaller quantities compared to the control (fruit before fermentation), representing an approximately 72% reduction in sucrose, a 96% reduction in glucose, and a 95% reduction in fructose. Literature suggests that around 60% of all sugars in coffee are consumed during fermentation [29], and the presence of residual fructose at the end of the fermentation process is commonly observed [14,37]. Low concentrations of sugars in coffee post-fermentation can contribute to the beverage’s sweetness [27].

Among the acids, citric, malic, and succinic acids are naturally present in the coffee fruit, while others are more prevalent post-fermentation. Across all treatments, higher concentrations of lactic acid were found in naturally fermented coffees (approximately 80 mg/g of coffee) compared to pulped coffees (approximately 60 mg/g of coffee), concentrations higher than those found at time zero (3.14 and 1.42 mg/g of coffee, for natural and pulped coffee, respectively). The presence of lactic acid in coffee is desirable, and some studies have used lactic acid bacteria as fermentation inoculants to produce higher-quality coffee [14]. Lactic acid is known to be one of the primary and most abundant organic acids produced in submerged coffee fermentation. In this study, solid-state fermentations also resulted in high concentrations of lactic acid [27,38,39].

Other acids were detected in all treatments, albeit in much smaller quantities compared to lactic acid. Acetic acid levels increased over the fermentation time in both natural and pulped coffee treatments (*p* < 0.05). Natural coffee treatments started with 2.5 ± 0.36 mg/g of coffee of acetic acid in the fruit and increased to 6.18 ± 1.25 mg/g for solid-state and 4.65 ± 1.5 mg/g for submerged after 96 h of fermentation. Pulped coffee treatments began with 1.06 ± 0.00 mg/g of coffee of acetic acid and rose to 2.62 ± 0.20 mg/g for solid-state and 3.64 ± 0.18 mg/g for submerged. After 96 h of fermentation, the natural coffee in solid-state process showed the highest production of acetic acid (6.18 mg/g of coffee) (*p* < 0.05).

For glycerol, an increase was observed only in natural coffee treatments (*p* < 0.05), with peak production in solid-state fermentation at 96 h (1.57 ± 0.9 mg/g of coffee) and in submerged fermentation at 72 h (1.14 ± 0.13 mg/g of coffee) when compared to the initial time (mean of 0.42 mg/g of coffee in natural and pulped coffee). No significant changes were noted over time for the pulped coffee treatments (Figure 2 and Table S2). Lower levels of glycerol in pulped coffees can be attributed to their processing method, which partially removes the exocarp and mesocarp, leading to a reduction in polysaccharide content. This decrease can limit glycerol formation, a metabolite derived from the degradation of sugars by yeasts, as suggested by various researchers [27].

Propionic and butyric acids, which are considered undesirable for beverage quality, were not detected in any of the treatments [34]. Jimenez et al. [40] also observed similar results, noting higher acid concentrations in natural coffees compared to pulped coffees, which they attribute to the higher sugar content in the mucilage of natural coffees.

Although all the treatments showed significantly higher values than the initial time (0.73 an 0.53 mg/g of coffee for natural e pulped coffee), alcohol production was highest in natural coffee in the solid-state process (62.05 mg/g of coffee after 96 h), twice as high as in natural coffee in the submerged process (31.3 mg/g of coffee) and approximately six times higher than in the pulped coffee treatments (17.25 mg/g of coffee). The difference between the natural coffee in the solid-state and submerged processes may be due to water acting as a solvent for alcohol molecules. The disparity between the treatments for natural and pulped coffee is attributed to the availability of sugars for fermentation.

The composition and concentrations of acids, sugars, and alcohols in coffees are key determinants of the final product’s sensory profile. The presence of citric acid, for instance, is valued in coffees for imparting an acidic profile with notes reminiscent of citrus fruits, like lemon and orange. Citric acid is produced under aerobic conditions through the Krebs cycle, and its detection in the initial phases of the fermentation process is expected due to the gradual consumption of oxygen and the subsequent transition to an anaerobic environment [41].

Malic acid, a natural constituent of the fruit, is associated with apple or green fruit flavors, enhancing the coffee’s profile complexity with fruity nuances. Bastian [42] notes that malic acid is a precursor to other acids, such as citric acid, and its presence was observed in this study only initially and after 24 h in treatments with natural coffee. Succinic acid, along with other acids, contributes to a pleasant acidity in fermented coffees [43].

Lactic acid, the most concentrated in all treatments, can provide a smoother and more rounded flavor profile, often associated with milky or yogurt-like notes, potentially imparting creaminess to the coffee [44]. Its elevated production can be attributed to the high sugar concentrations available in the fermentation processes, which are generated by lactic acid bacteria metabolizing hexoses and pentoses [41]. Acetic acid can be produced by the oxidation of ethanol under aerobic conditions or by acetogenic bacteria in anaerobic environments. Although it adds notes of acidity to coffee, excessive presence can lead to vinegary or undesirable flavors [20,44]. Glycerol contributes to the body of the beverage, enhancing creaminess and affecting mouthfeel and perceived texture; plays a crucial role in beverage stability, particularly in retaining aromas and flavors; and contributes to the Maillard reaction [27,45]. Ethanol is responsible for imparting alcoholic notes to coffee and is important for the beverage’s viscosity, also acting as a solvent for volatile compounds [27].

**Figure 3:**
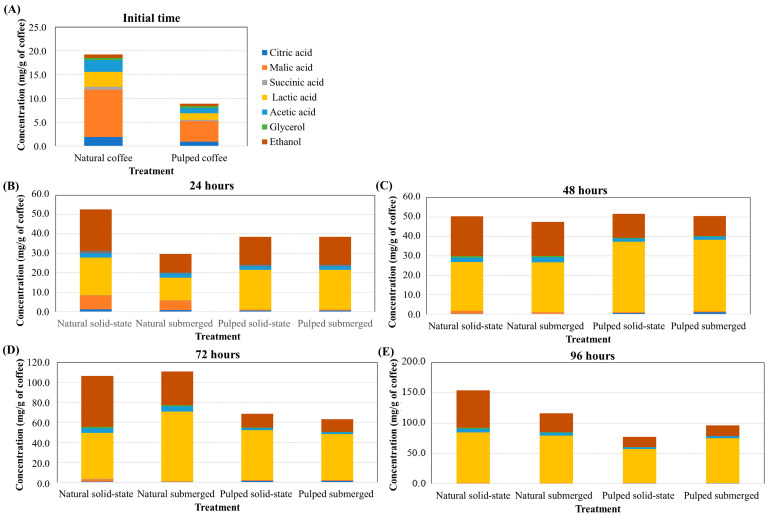
Production of acids and alcohols over the 96 h fermentation process of Catiguá MG2 coffee (data presented in mg/g of dry coffee mass). The treatments evaluated were natural and pulped coffee fruits subjected to SIAF in solid-state and submerged processes at initial time (**A**), 24 h (**B**), 48 h (**C**), 72 h (**D**), and 96 h (**E**).

In the production of specialty coffees, it is crucial to assess which fermentation process is the most effective in producing specific acids and alcohols as well to determine determining the necessary fermentation time for these compounds to reach desired concentrations. While coffee fruit naturally contains small amounts of acids and alcohols, these concentrations are initially very low. Prior to fermentation, in both natural and pulped coffee fruits, malic acid has the highest concentration (9.91 and 4.31 mg/g of coffee for natural and pulped coffee, respectively), followed by lactic (3.14 and 1.41 mg/g of coffee), acetic (2.46 and 1.06 mg/g of coffee), and citric acids (1.90 and 0.94 mg/g of coffee) (Figure 3). At 24 h of fermentation, there is an increase in lactic acid concentration in the pulped treatments (20.45 mg/g of coffee) and a significant increase in alcohol content in the natural coffee solid-state process (21.22 mg/g of coffee), while malic acid is still detectable in treatments with natural coffee. After 48 h, the production of lactic acid continues to be more pronounced in the pulped coffee treatments (36.50 mg/g of coffee), and at 72 (58.00 mg/g of coffee for natural, and 48.11 mg/g of coffee for pulped) and 96 h (80.76 mg/g of coffee for natural and 65.00 mg/g of coffee for pulped), the natural coffee treatments exhibit higher concentrations of this acid.

The concentration of ethanol was consistently higher in all periods in the natural coffee treatments, particularly in the solid-state process. Acetic acid was also detected in greater quantities in all periods in the natural coffee treatments, while no significant differences were observed in the other acids (*p* > 0.05) (Figure 3). These data are crucial for producers to make informed decisions when selecting the fermentation process based on the desired sensory characteristics for specialty coffee consumers. 

**Figure 4:**
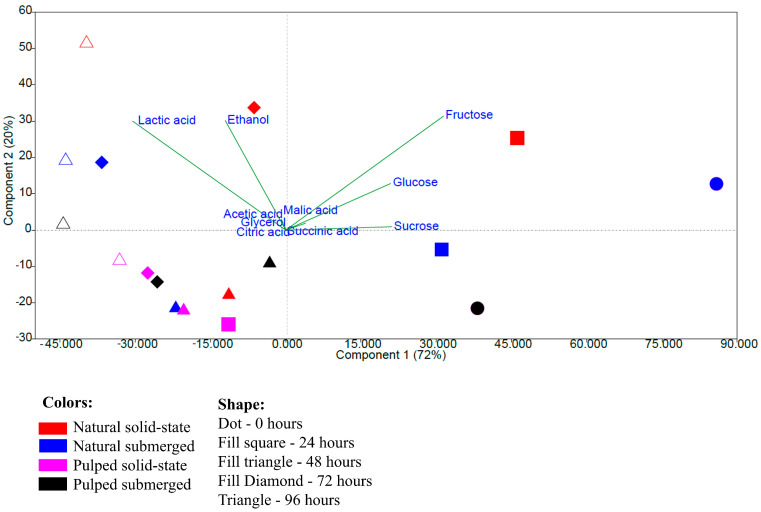
Principal component analysis based on data from the quantification of sugars, acids, and alcohols during the 96 h fermentation process of Catiguá MG2 coffee. The treatments evaluated were natural and pulped coffee fruit subjected to SIAF in solid-state and submerged processes.

The principal component analysis performed with the data on the quantification of sugars, acids, and alcohols shows a more pronounced grouping related to the fermentation time rather than the type of processing. The variables fructose, glucose, and sucrose were responsible for the grouping at times 0 and 24 h, and lactic acid and ethanol for the groupings at 72 and 96 h, while other variables did not significantly influence the groupings. Principal components 1 and 2 together explained 92% of the observed variation (Figure 4). The results indicate that the initial samples and those at 24 h have greater similarity to each other, as do those at 72 and 96 h, based on quantification performed via high-performance liquid chromatography.

The mucilage surrounding the coffee beans is rich in sugars, primarily sucrose, glucose, and fructose. During fermentation, microorganisms, such as bacteria and yeasts, metabolize these sugars, converting them into various products, such as acids, alcohols, and other compounds, in a metabolic process that is essential for the development of the coffee’s flavor profile. The microbial diversity involved in the fermentation process is related to different factors, such as the variety of coffee and its natural microbiota, the characteristics of the soil and climate in the region, and the method of post-harvest processing among others. Several genera of bacteria and yeasts have already been described in the coffee fermentation process, the most commonly found being the genera *Bacillus*, *Lactobacillus*, *Acinetobacter*, *Arthrobacter,* and *Weissella* representing the bacteria and the genera *Pichia*, *Saccharomyces*, *Rhodotorula*, *Candida*, *Hanseniaspora,* and *Kluyveromyces* representing the yeasts.

Different microorganisms can produce different acids, such as lactic, acetic, and citric acids, contributing to the complexity and uniqueness of the flavor profile. The presence and proportion of these acids in the coffee beverage are key determinants for specialty coffees to exhibit distinct and appealing sensory profiles for various audiences [17,21,27,34,46].

### 3.4. Sensorial Analysis

**Figure 5:**
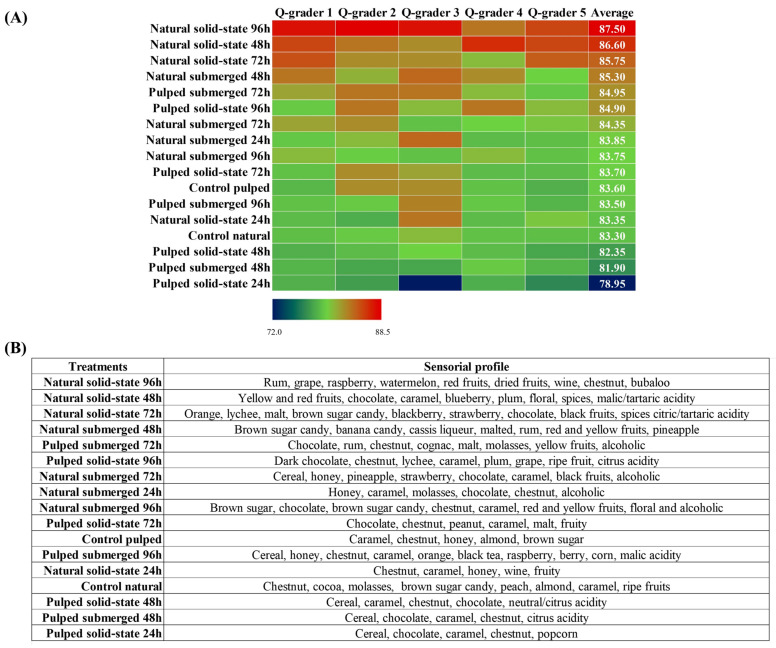
Classification of coffees obtained over the 96 h fermentation process. The treatments evaluated were natural and pulped coffee fruit subjected to SIAF in solid-state and submerged processes. The evaluation was conducted by Q-graders (*n* = 5), and scores (**A**) and sensory profiles (**B**) were assigned.

In the sensory analysis, coffees derived from various fermentative processes were evaluated by Q-Graders licensed by the Coffee Quality Institute (CQI) using the methodology established by the Specialty Coffee Association (SCA). Compared to the controls of each variant, either natural or pulped, the scores obtained ranged from 78.95 to 87.50. With the exception of the pulped coffee solid-state treatment at 24 h, all other treatments, including controls, were classified as specialty coffees (score above 80) (Figure 5A). Among the eight treatments with pulped coffee, four had scores lower than their respective controls (control pulped = 83.60). The three highest average scores were observed in the natural coffees in solid state at durations of 96, 48, and 72 h, achieving scores of 87.50 ± 0.9, 86.60 ± 0.8, and 85.75 ± 0.9 (mean ± standard deviation), respectively. Overall, natural coffees resulted in fermented beverages of superior quality compared to pulped coffees, surpassing the scores of their respective controls (83.30). These results suggest that fermentation is a crucial post-harvest step capable of enhancing the quality of coffee, corroborating previous studies indicating the Catiguá variety as conducive to the production of high-quality beverages. Additionally, solid-state fermentation proved to be an effective technique in reducing water consumption by up to 89%, particularly when employed in the processing of natural coffee, further saving water by omitting the depulping stage [47].

Besides enhancing the quality of coffees, the fermentative process facilitates a modification in the sensory profile of the beverage, varying according to the applied processing method. Treatments with similar scores may exhibit distinct sensory profiles, offering a diversity of flavors to satisfy different consumer preferences. The main sensory attributes identified in the treatments evaluated in this study include nuances of caramel, nut, and chocolate as well as alcoholic variations, although each treatment presents its unique characteristics (Figure 5B). Research conducted by Jimenez et al. [40] investigating the self-induced anaerobic fermentation of Topázio variety coffee also reported similar sensory profiles, highlighting notes of citrus fruits, caramel, honey, chocolate, and nuts for natural coffees and citrus fruits, nuts, chocolate, caramel, and nuts for pulped coffees. The specific sensory profile for each treatment, as described by the evaluators, is summarized in Figure 5B. A marked presence of cereal notes is noted in the treatments with pulped coffee, which obtained the lowest scores. The sensory profile of the coffee with the highest evaluation, natural coffee in the solid-state process at 96 h, features notes of rum (alcoholic), red fruits, and watermelon, differing from the notes of chocolate, caramel, and brown sugar candy identified in the 48 and 72 h treatments. The alcoholic sensory presence, as identified by the evaluators, aligns with the amounts of ethanol observed in the treatments, particularly those with natural coffee. Furthermore, the evaluators also described the presence of citric and malic acidity, as well as notes of nuts and coconut, corresponding to the production of citric, malic, and lactic acids, respectively, also detected in liquid chromatography analyses. 

**Figure 6:**
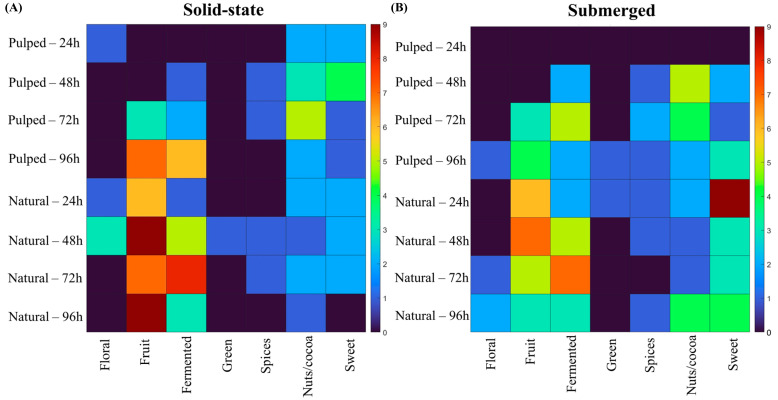
Sensory attributes of coffees obtained over the 96 h fermentation process, according to the SCA sensory wheel. The treatments evaluated were natural and pulped coffee fruit subjected to SIAF in solid-state (**A**) and submerged processes (**B**).

When correlating the sensory attributes designated by the evaluators for each treatment with the flavor wheel proposed by the Specialty Coffee Association (SCA), we observe the results shown in Figure 6.

In the natural coffee treatments in solid state at 24 and 48 h and in the pulped coffee treatment at 24 h, floral notes were observed. In submerged fermentation treatments, the floral character was more prevalent towards the end of the process, at 72 and 96 h for natural coffee and at 96 h for pulped coffee. The fruity flavor was more pronounced in natural coffee treatments, particularly in the solid-state process at 48 and 96 h. The perceptions of nuts and coconut were more accentuated in the pulped coffee treatments at 48 and 72 h, corroborating the results found for lactic acid production.

**Figure 7:**
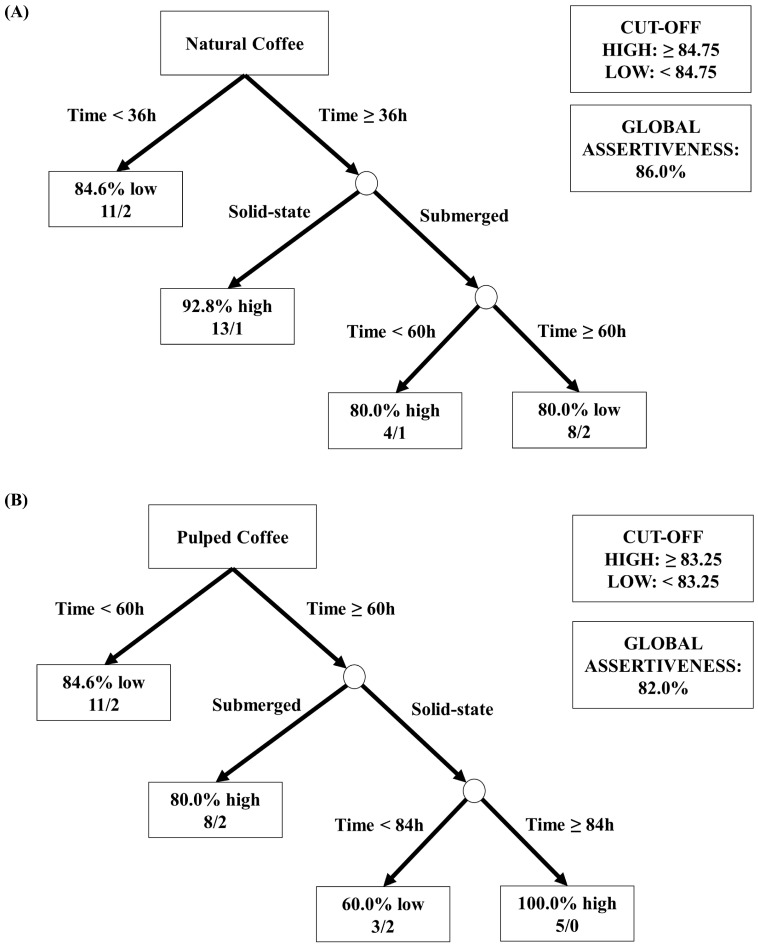
Decision tree representation based on the sensory scores obtained in the classification of coffee beverages fermented for 96 h. The treatments evaluated were natural and pulped coffee fruit subjected to SIAF in solid-state and submerged processes. The trees represented are for natural coffee (**A**) and pulped coffee (**B**).

As depicted in Figure 6, different coffee processing methods (natural and pulped), fermentation pathways (solid-state or submerged), and fermentation durations result in distinct sensory profiles, each with unique characteristics. To aid in data interpretation and simplify decision-making in selecting strategies for producing specialty coffees through fermentation, visual decision trees were developed using machine learning algorithms. These trees aim to provide a clear understanding of the most effective methodologies identified in the experiments (Figure 7).

Figure 7A shows the analysis using data from natural coffees, with an overall accuracy of 86%. The threshold for high sensory notes was set as equal to or greater than 84.75, and notes below this value were classified as low. The first node (root node) considered the “Time” variable as the most significant division factor, indicating that for durations below 36 h, the majority of results were classified as low (84.6%). For experiments with durations above 36 h, in the subsequent node, the “Processing Type” variable (submerged or solid-state SIAF) was used for division. It was observed that the best sensory results were obtained from the process with natural coffee in solid state and a fermentation duration above 36 h, where 93% of the samples achieved scores equal to or above 84.75 points by the SCA. For the process with natural coffee in submerged fermentation and duration above 36 h, the subsequent division considered a threshold of 60 h. This division revealed that for natural coffee in the submerged process with a duration of 48 h, the sensory evaluation was high, while for durations above 60 h, the evaluation was low.

In Figure 7B, the data from the sensory analyses of pulped coffees showed an overall accuracy of 82%. The threshold for high sensory notes was established as equal to or greater than 83.25, and notes below this value were classified as low. Similarly, the first node (root node) considered the “Time” variable as a critical factor for differentiating the results. Thus, it was observed that the evaluations of coffee beverages obtained in treatments with durations below 60 h were predominantly low (84.6% of the experiments below 83.25 points on the SCA scale). For durations above 60 h, a subsequent node separated the samples based on the “Processing Type”, revealing that beverages from submerged treatments received high sensory scores in 80% of the cases. For solid-state treatments, the “Time” variable was again considered to differentiate the results into high and low. It was found that beverages from solid-state fermentations achieved the best results with durations above 84 h for pulped coffee. 

The results obtained from the quantitative analyses of sugars, acids, and alcohols; the ratings and sensory profiles assigned by the Q-graders; and the decision trees suggest that the best approach for producing specialty coffees using the Catiguá MG2 variety is through the natural coffee fermentation in a solid state. A duration of 96 h has been identified as optimal for effective fermentation without fostering the formation of undesirable acids, although a period of 48 h has already shown the capability to produce high-quality coffee with a reduced processing time. 

## 4. Conclusions

The Catiguá coffee variety, an Arabica species, responds well to the fermentative process, maintaining the physical characteristics of the green bean (firmness and hardness), resulting in coffees classified as special with scores surpassing the controls. Additionally, this variety possesses an effective natural microbiota for fermenting the mucilage sugars, producing various acids and alcohols, notably lactic acid and ethanol.

All the fermentative processes evaluated, including natural and pulped coffee subjected to solid-state and submerged fermentation (SIAF), exhibited different sensory profiles among themselves and at various fermentation times. The best results were achieved using natural coffee fermentation in a solid-state process with durations above 24 h, although fermentation also proved effective in enhancing the quality of pulped coffees. This study can contribute to a better understanding and knowledge of the influence of different fermentative processes in the production of specialty coffees and promote better process control conditions and, consequently, greater reproducibility of results.

## Data Availability

The original contributions presented in the study are included in the article, further inquiries can be directed to the corresponding author.

## Supplementary Materials

The following supporting information can be downloaded at: https://www.mdpi.com/article/10.3390/foods13050653/s1, Table S1: Values of texture parameters (strength and hardness) observed for the green bean during the 96 h fermentation process of Catiguá MG2 coffee. The evaluated treatments were natural and pulped coffee subjected to SIAF in solid-state and submerged processes. The data presented are the means (n = 3) and their respective standard deviations. Means followed by the same letter indicate no statistical difference between treatments (*p* > 0.05); Table S2: Values obtained from the quantification of sugars, acids, and alcohols in coffees processed over 96 h of fermentation (data presented in mg/g of dry coffee mass). The treatments evaluated were natural and pulped coffee subjected to SIAF in solid-state and submerged. The data presented are averages (n = 3) followed by their respective standard deviations. Distinct lowercase letters following the averages indicate significant statistical differences between different fermentation times for the same treatment, and uppercase letters indicate differences between different treatments in the same period (*p* < 0.05).
