# Supplementary material for: Enhancing Sensory Quality of Coffee: The Impact of Fermentation Techniques on Coffea arabica cv. Catiguá MG2
Source: Foods. 2024 Feb 21;13(5):653. doi: 10.3390/foods13050653 (PMC10931400; doi:10.3390/foods13050653)
Supplement: Supplementary file 1 [file foods-13-00653-s001.zip › foods-2757043-supplementary.pdf]

Table S1. Values of texture parameters (strength and hardness) observed for the green bean during the 96-hour fermentation process of Catiguá MG2 coffee. The evaluated treatments were natural and pulped coffee subjected to SIAF in solid-state and submerged processes. The data presented are the means (n=3) and their respective standard deviations. Means followed by the same letter indicate no statistical difference between treatments (p>0.05).

**Fermentation Time**	**Treatment**	**Strength (g)**	**Hardness (g)**
**24 hours**	Natural submerged	48124,00±1016,75 a	48498,30±966,78 a
	Natural solid-state	50239,13±7871,93 a	50922,93±6689,45 a
	Pulped submerged	49123,12±3547,12 a	50882,10±1030,12 a
	Pulped solid-state	49192,48±4315,54 a	50182,86±4570,99 a
**48 hours**	Natural submerged	51054,11±2791,25 a	51906,09±2330,16 a
	Natural solid-state	52559,57±1412,66 a	53547,22±1402,13 a
	Pulped submerged	47018,29±3665,35 a	47899,06±3350,87 a
	Puped solid-state	52733,49±2721,71 a	52771,15±2481,57 a
**72 hours**	Natural submerged	49883,76±8138,69 a	51085,65±7097,66 a
	Natural solid-state	45940,56±2133,5 a	46911,46±2085,89 a
	Pulped submerged	45492,26±4101,26 a	45872,98±3748,29 a
	Puped solid-state	47973,15±4114,45 a	48858,02±3040,12 a
**96 hours**	Natural submerged	52988,19±4884,77 a	53802,89±4821,06 a
	Natural solid-state	52932,35±4667,40 a	53543,64±4235,99 a
	Pulped submerged	49185,73±3733,17 a	50317,57±3406,32 a
	Puped solid-state	48082,02±5563,00 a	49290,03±4663,18 a
**Control**	Natural Control	50662,91±3650,41 a	51839,20±3610,82 a
	Pulped Control	48456,50±2473,97 a	49157,73±2128,89 a

Table S2. Values obtained from the quantification of sugars, acids, and alcohols in coffees fermented for 96 hours (data presented in mg of acid.g^{-1} of dry coffee mass). The treatments evaluated were: natural and pulped coffee subjected to SIAF in solid-state and submerged. The data presented are averages (n=3) followed by their respective standard deviations. Significant statistical differences between different fermentation times for the same treatment are indicated by distinct lowercase letters, and differences between different treatments in the same period are indicated by uppercase letters (p<0.05).

		**Citric acid**		**Malic acid**		**Succinic acid**		**Lactic acid**		**Acetic acid**	
	**Time**	Average	SD	Average	SD	Average	SD	Average	SD	Average	SD
**Natural Solid-state**	0 hours	1,91 aA	0,60	9,91 aA	1,89	0,61 aA	0,16	3,14 cA	1,19	2,46 bcA	0,39
	24 hours	1,13 abA	0,32	7,20 aA	1,22	0,15 bcA	0,02	19,41 cA	2,94	2,20 cA	0,50
	48 hours	0,25 cBC	0,11	1,50 bA	0,29	0,05 ccA	0,01	25,02 bcA	6,24	2,30 cA	0,67
	72 hours	0,62 bcAB	0,14	2,33 bA	0,62	0,45 abA	0,24	46,11 bB	11,19	4,63 abA	1,09
	96 hours	0,25 cC	0,03	1,29 bA	0,08	0,08 cA	0,01	82,84 aA	13,79	6,18 aA	1,25
**Natural Submerged**	0 hours	1,90 aA	0,60	9,91 aA	1,89	0,61 aA	0,16	3,14 bA	1,18	2,46 bA	0,39
	24 hours	0,87 bA	0,23	4,79 bB	0,39	0,13 bA	0,01	11,72 bB	2,29	2,02 bA	0,25
	48 hours	0,29 bC	0,01	0,73 cB	0,07	0,05 bA	0,01	25,75 bA	2,19	2,53 bA	0,29
	72 hours	0,35 bB	0,04	0,56 cB	0,08	0,07 bB	0,01	69,85 aA	5,63	5,27 aA	0,35
	96 hours	0,37 bBC	0,08	0,44 cB	0,13	0,01 bB	0,05	78,69 aA	4,33	4,65 aAB	1,51
**Pulped Solid-state**	0 hours	0,94 abA	0,22	4,30 aB	1,45	0,24 aB	0,11	1,41 cA	0,189	1,06 bB	0,33
	24 hours	0,58 bA	0,09	0,45 bC	0,06	0,02 bB	0,00	20,45 bcA	2,64	1,83 abA	0,31
	48 hours	0,68 abAB	0,12	0,36 bB	0,07	0,00 bB	0,00	36,20 abA	4,62	1,66 abA	0,61
	72 hours	1,34 aA	0,48	0,71 bB	0,26	0,00 bB	0,00	50,13 aAB	5,84	2,18 aB	0,31
	96 hours	0,95 abA	0,18	0,45 bB	0,08	0,00 bB	0,00	56,12 aA	5,28	2,62 aB	0,21
**Pulped Submerged**	0 hours	0,94 aA	0,22	4,30 aB	1,45	0,24 aB	0,11	1,41 cA	0,18	1,06 cB	0,33
	24 hours	1,08 aA	0,21	1,50 bC	0,04	0,00 bB	0,00	21,29 bA	3,21	1,20 cA	0,19
	48 hours	1,04 aA	0,30	0,49 bB	0,13	0,00 bB	0,00	36,79 bA	2,62	1,59 bcA	0,20
	72 hours	1,35 aA	0,45	0,67 bB	0,22	0,00 bB	0,00	46,10 bB	9,35	1,93 bB	0,21
	96 hours	0,59 aB	0,11	0,41 bB	0,05	0,00 bB	0,00	73,90 aA	6,60	3,64 aAB	0,18
		**Sucrose**		**Glucose**		**Fructose**		**Glycerol**		**Ethanol**	
	**Time**	Average	SD	Average	SD	Average	SD	Average	SD	Average	SD
**Natural Solid-state**	0 hours	59,75 aA	8,48	49,47 aA	6,27	82,28 aA	11,46	0,46 cA	0,40	0,73 cA	0,67
	24 hours	13,41 bA	4,99	42,41 aA	5,43	73,40 aA	9,61	1,25 abA	0,04	21,23 bA	2,12
	48 hours	0,54 cC	0,22	11,48 bA	1,55	23,02 bA	4,91	0,80 bcA	0,22	20,55 bA	4,84
	72 hours	2,86 bcB	0,85	17,96 bA	6,35	43,97 bA	13,57	1,35 abA	0,40	51,18 aA	9,62
	96 hours	0,27 cB	0,10	9,36 bA	0,51	33,56 bA	3,70	1,57 aA	0,09	62,06 aA	10,56
**Natural Submerged**	0 hours	59,74 aA	8,48	49,47 aA	6,27	82,27 aA	11,46	0,46 bA	0,40	0,73 dA	0,67
	24 hours	14,83 bA	4,41	25,97 bB	0,41	45,53 bB	0,37	0,64 abB	0,04	9,51 cdC	1,13
	48 hours	1,13 cBC	0,09	3,79 cB	0,58	13,28 cB	1,24	0,79 abA	0,12	17,52 bcAB	1,95
	72 hours	2,97 cAB	0,44	2,93 cB	0,82	17,92 cB	2,13	1,14 aA	0,13	33,85 aB	4,00
	96 hours	2,29 cB	0,74	1,76 cB	0,83	14,29 cB	4,59	0,88 abB	0,33	31,27 abB	11,20
**Pulped Solid-state**	0 hours	29,36 aB	5,55	22,57 aB	6,08	37,02 aB	11,44	0,38 bA	0,20	0,53 bA	0,09
	24 hours	2,25 bB	0,55	2,30 bC	0,53	7,06 bC	1,13	0,82 bB	0,17	14,34 aB	1,83
	48 hours	4,19 bAB	0,64	0,76 bC	0,27	3,13 bC	1,18	0,37 bB	0,16	12,39 aAB	5,20
	72 hours	8,77 bA	2,17	1,75 bB	0,87	3,75 bB	1,12	0,28 bB	0,02	13,87 aC	2,67
	96 hours	8,07 bA	1,95	0,83 bB	0,09	2,18 bC	0,16	0,24 bC	0,01	17,03 aB	1,56
**Pulped Submerged**	0 hours	29,36 aB	5,55	22,57 aB	6,08	37,02 aB	11,44	0,38 aA	0,20	0,53 cA	0,09
	24 hours	10,42 bA	2,56	2,12 bC	0,23	7,02 bC	0,98	0,32 aB	0,05	8,12 bC	1,23
	48 hours	7,43 bA	2,57	1,12 bC	0,20	3,20 bC	0,44	0,30 aB	0,07	10,34 bB	1,03
	72 hours	6,25 bAB	3,81	2,32 bB	0,23	4,08 bB	0,57	0,23 aB	0,01	12,95 bC	2,62
	96 hours	5,42 bA	0,80	0,74 bB	0,07	2,06 bC	0,13	0,11 aC	0,03	17,48 aB	0,16
